# Minimally Invasive Two-Staged Surgery in the Treatment of Large Cystic Lesions of the Jaw

**DOI:** 10.3390/healthcare9111531

**Published:** 2021-11-10

**Authors:** Andreea Irimia, Liliana Moraru, Diana Alina Ciubotaru, Constantin Caruntu, Alexandru-Titus Farcasiu, Ana Caruntu

**Affiliations:** 1 Department of Oral and Maxillofacial Surgery, “Carol Davila” Central Military Emergency Hospital, 010825 Bucharest, Romania; irimia.andreeaioana@yahoo.com (A.I.); liliana.moraru@yahoo.com (L.M.); ciubotarudianaalina@gmail.com (D.A.C.); ana.caruntu@gmail.com (A.C.); 2 Department of Oral and Maxillofacial Surgery, Faculty of Dental Medicine, “Titu Maiorescu” University, 031593 Bucharest, Romania; 3 Department of Physiology, “Carol Davila” University of Medicine and Pharmacy, 050474 Bucharest, Romania; 4 Department of Dermatology, “Prof. N.C. Paulescu” National Institute of Diabetes, Nutrition and Metabolic Diseases, 011233 Bucharest, Romania; 5 Department of Removable Prosthodontics, “Carol Davila” University of Medicine and Pharmacy, 020021 Bucharest, Romania

**Keywords:** minimally invasive, mandibular cyst, marsupialization, reconstruction

## Abstract

Background: Cystic lesions of the jaw are commonly found in clinical practice. Large, expansive cysts raise challenges for the clinician from both diagnostic and surgical perspectives. The aim of our work is to present a combined, two-staged surgical approach in histologically confirmed non-aggressive cystic lesions of the jaw. Methods and Results: We report the case of an extensive mandibular cyst, associating a high risk of bone fracture, that is treated in the initial stage by cystic decompression through marsupialization with concomitant histological diagnostic confirmation, followed in the second stage by radical excision and mandibular reconstruction with titanium mesh, with the purpose of prevention for oro-cystic chronic fistula formation. Conclusions: Large odontogenic mandibular cysts imply a meticulously conducted assessment and treatment. Marsupialization should be taken into consideration for the treatment of large cystic lesions, followed by secondary enucleation, with minimal risks for the patient. The soft tissue healing process can be optimized with the use of titanium meshes, as an alternative for other reconstructive techniques, in the management of large cystic lesions.

## 1. Introduction

Odontogenic cysts are the most common cause of jaw radiolucent lesions and include a wide range of pathological entities. Radicular or residual cysts, keratocysts, and dentigerous cysts represent the vast majority of odontogenic lesions found in clinical practice [1,2]. Radicular and residual lesions, known as inflammatory cysts, usually have limited growth, with no major consequences on mandibular resistance. However, in isolated cases, these cysts can have aggressive growth patterns with extensive bone resorption, that can significantly impair mandibular resistance [3,4]. The intracystic increased fluid pressure has been shown to influence the dimensions of the lesions, especially in the initial stages of cystic development [5,6]. This increased pressure is most probably caused by epithelial cells desquamation from the cystic walls, releasing their intracellular content into the cystic cavity. This leads to an increased osmotic pressure, which in consequence induces an elevation of the intracystic hydrostatic pressure [7]. An intense inflammatory activity within the cystic membrane, specific for residual and radicular cysts, has also been considered to play an important role in cystic growth and peripheral bone resorption [8]. However, in most cases, these mechanisms coexist and contribute to lesion expansion and peripheral bone resorption, leading to the development of giant cysts associated with an impaired mandibular resistance, prone to fracture.

It is very important to differentiate radicular or residual cysts from other bone lesions [9] that can exhibit similar clinical and imagistic characteristics, such as unicystic ameloblastoma or odontogenic keratocyst [10,11]. These lesions have distinct clinic-pathological behavior, associating high rates of recurrence, and imply completely different therapeutic approaches. Thus, a diagnostic biopsy is strongly recommended before any radical treatment, in order to rule out aggressive bone lesions. Primary excision of large mandibular cysts that have severely undermined the mandibular bone structure is not feasible due to the high risk of jaw fractures, challenging to treat considering the lack of bony tissue for stable plating [4]. The risk–benefit balance in patients with giant cystic lesions that do not exhibit other aggressiveness features does not favor extensive, complex reconstructive surgery in most cases, due to the increased incidence of perioperative complications [12,13]. Cystic decompression through marsupialization followed by second stage radical excision can be a valid therapeutic option in these patients, providing several advantages: cystic decompression with simultaneous biopsy for diagnostic confirmation, minimally invasive surgery conducted under local anesthesia, outpatient care or reduced hospitalization time, and rapid post-surgical recovery [14]. The main disadvantage is the temporary presence of an oro-cystic fistula during the interval between the two surgeries, which requires daily care and frequent follow-up visits that might be challenging for some patients. Another potential complication after the two-staged approach—marsupialization and excision—is the persistent oro-cystic fistula after secondary surgery, which can sometimes be difficult to manage.

The present case report depicts the clinical characteristics and the therapeutic approach of a massive residual mandibular cyst, treated through a two-stage approach: initial cystic decompression with simultaneous diagnostic biopsy, followed by secondary radical excision. In order to reduce the risk of persistent oro-cystic fistula, bone reconstruction with titanium mesh was performed for the mandibular fenestration, thus providing solid support for the adjacent soft tissues. The particularities of this case are related to an atypical clinical behavior, with an aggressive growth pattern for an otherwise inoffensive clinical entity, and the therapeutic approach that allowed an accurate diagnosis and simultaneous treatment, through minimally invasive techniques, leading to optimal therapeutic results and no complications.

## 2. Case Report

**Figure 1:**
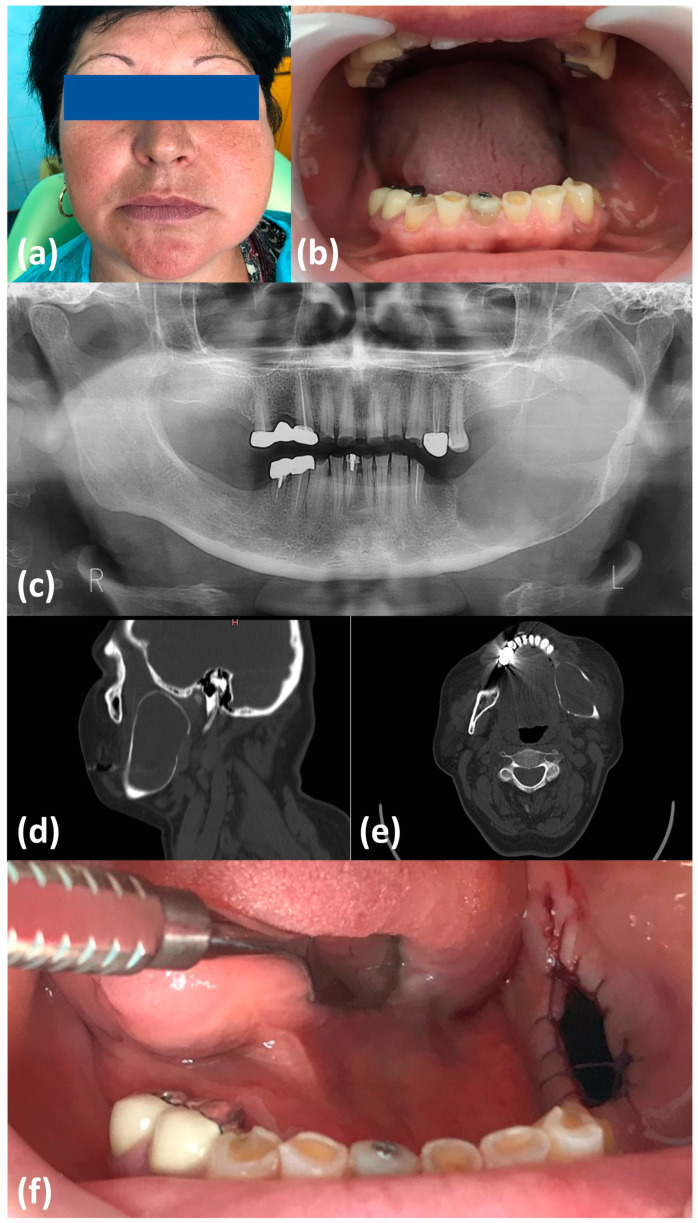
Clinical and imagistic aspect at presentation. (**a**) Extraoral aspect; (**b**) intraoral aspect; (**c**) preoperative panoramic X-ray; (**d**) preoperative native CT scan: sagittal view; (**e**) axial view; (**f**) intraoral aspect after marsupialization of the cyst.

In February 2019, a 53-year-old female patient was admitted to the Department of Oral and Maxillofacial Surgery, “Dr. Carol Davila” Central Military Hospital, Bucharest, with an important facial asymmetry on the left side, caused by the presence of a swelling affecting the middle and lower thirds of the face, that was noticed by the patient about three months before admission (Figure 1a). The lesion was asymptomatic, painless at palpation, overall of hard consistency except for small renitent areas at the intraoral examination. The patient reported no history of infectious exacerbations. The skin lining the affected area did not present any changes in color, temperature, and turgor and did not associate any motor or sensory deficit. The patient did not report any previously known conditions and did not take any chronic medication. General clinical and paraclinical assessment of the patient did not reveal any other significant pathological changes.

A detailed dental history from the patient revealed having multiple teeth extractions in the third quadrant a few years before with no other history of swelling and pain until now. Intraoral examination showed a bilateral terminal edentulous lower arch, severe attrition, and a bicortical swelling on the left side of the mandible. The oral mucosa lining was of normal appearance, except for a small central area of translucent color, corresponding to the previously mentioned renitent area (Figure 1b).

The initial panoramic X-ray showed a very large radiolucent lesion, extended from the region of the first molar–the angle of the mandible–vertical ramus–coronoid process, leaving intact only the left condyle. The contour of the mandible was altered, and areas of peripheral sclerosis were present. The projection of the left mandibular canal was displaced downwards. There were no teeth, impacted or erupted, in relation to the lesion (Figure 1c).

Native computed tomography (CT) revealed the presence of a large bony cavity extended in the left body and ramus of the mandible, measuring 80 mm × 35 mm in size. The cavity presented several areas of complete bone loss, with fenestration of the lingual and basilar cortical bone, deviation of the mandibular canal, and complete disruption of the superior wall of the canal (Figure 1d,e).

Considering the major bone loss with a very high risk of mandibular fracture and the unknown diagnosis, we initiated a minimally invasive two-staged approach to treat this patient. In the first stage, the decompression of the cyst through marsupialization was performed. Under local anesthesia, we designed an ellipsoidal incision with the excision of an area of oral mucosa and adjacent cystic membrane, thus creating a fenestration of 1.5 per 1 cm between the cystic lesion and oral cavity. The cystic content, which had a greyish transparent aspect with small white inclusions, was evacuated and the edges of the oral mucosa were sutured to the cystic lining, using a technique of continuously running suture, thus preventing wound closure. The removed cystic tissue was sent for pathological assessment, which described the presence of inflamed non-keratinized squamous epithelium with cholesterol clefs and fibro-vascular tissues, suggestive for a residual cyst (Figure 1f).

**Figure 2:**
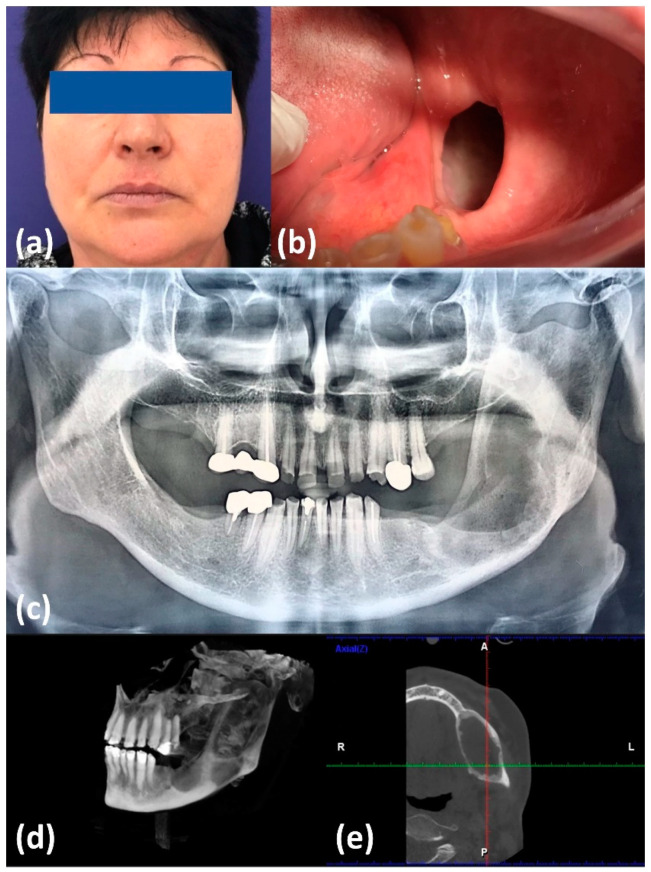
Clinical and imagistic aspect at 3 months after marsupialization. (**a**) Extraoral aspect; (**b**) intraoral aspect; (**c**) panoramic X-ray; (**d**) CBCT with 3D reconstruction; (**e**) CBCT axial view.

The patient was instructed on daily oral hygiene routine: tooth brushing and rinsing after every meal, as well as local chlorhexidine gluconate gel application for a period of three weeks, and was called every two weeks for follow-up visits. After 3 months, the patients’ appearance improved significantly, with almost complete restoration of facial symmetry (Figure 2a,b) The imagistic examination—panoramic X-ray and cone beam computed tomography (CBCT)—evidenced an important reduction of the cystic dimensions, with the restoration of the normal mandibular contour and significant circumferential bone apposition, as a witness for active bone regeneration (Figure 2c–e).

**Figure 3:**
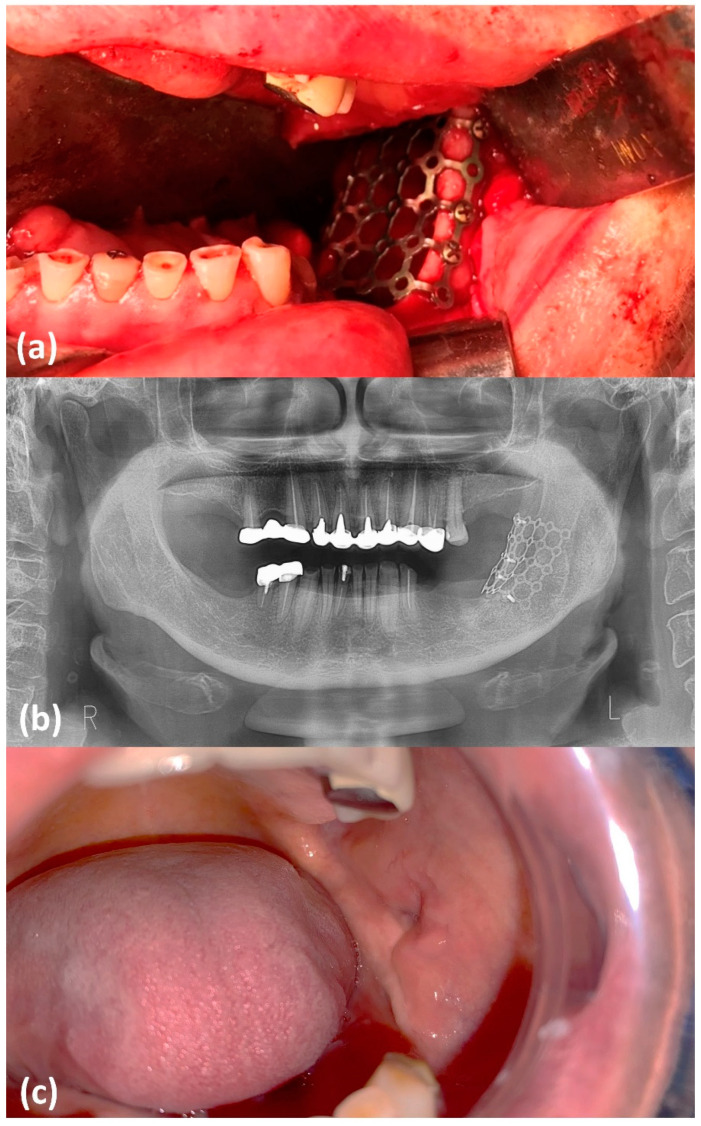
Intraoperative aspect from second-stage surgery and imagistic aspect at 12 months follow-up (**a**) intraoperative aspect depicting the titanium mesh fixed with titanium screws; (**b**) panoramic X-ray at 12 months follow-up; (**c**) intraoral postoperative aspect at 12 months follow-up.

At 6 months, the decompression effects were stable, and the patient underwent the second stage of the treatment. Under general anesthesia, radical cystic excision was performed. The bone defect corresponding to the marsupialization window was reconstructed with a titanium mesh, fixed with screws, in order to prevent the formation of a chronic oro-cystic fistula (Figure 3a). Postoperative care included local cold packs, soft diet, daily oral rinses with chlorhexidine gluconate mouthwash, antibiotic prophylaxis (ceftriaxone injection 1 g TID for 5 days), and anti-inflammatory drugs (ketoprofen injection 100 mg TID for 3 days). The sutures were removed two weeks after surgery, revealing a complete wound closure with no mesh exposure. At 12 months follow-up visit, clinical and imagistic assessments revealed a postoperative mucosa scar with no evidence of fistula formation and appropriate bone apposition, comparable to the contralateral healthy side of the mandible (Figure 3b,c).

## 3. Discussion

The surgical management of cystic lesions involving the jaws has become progressively less invasive and the choice for a certain technique is influenced by cystic characteristics, such as localization, dimension, or rate of recurrence [15,16]. In small lesions, the diagnosis and definitive treatment can be carried out in one-stage surgery, consisting of radical excision and pathology examination of the specimen to rule out an aggressive recurrent lesion, without high risks for complications [17]. However, in large cystic mandibular lesions, one-stage radical surgery is rarely an option due to the increased risks of intraoperative and postoperative complications [18]. The main risk in treating giant cystic lesions of the mandible with a one-stage approach is for an immediate or secondary bone fracture caused by the reduced bone resistance [19]. Furthermore, these fractures are challenging to treat due to the lack of normal bone structure and usually require complex reconstruction techniques in order to restore mandibular continuity, with additional risks for the patient and financial burden for the healthcare system. Reconstructive surgery using avascular bone grafts or even free flap grafts can be used as an alternative treatment option for these lesions. However, these complex surgeries imply additional risks, like donor site morbidity, potential loss of the flap or graft, or visible scars [20,21,22]. In our presentation, we emphasize the role and benefits of a minimally invasive technique in the treatment of large mandibular cystic lesions associated with proactive management of potential complications. Our conservative, two-staged surgery, consisting of initial marsupialization with simultaneous diagnosis, followed by radical resection and reconstruction of the defect with a titanium mesh implant, provided an optimal therapeutic approach for an otherwise aggressively growing lesion with almost complete mandibular resorption. Decompression of the cysts has been long documented with beneficial effects on bone regeneration [23,24,25]. In our case, within three months, the dimensions of the lesion have reduced by more than 50%, and significant bone regeneration could be evidenced through imagistic assessment, with new bone apposition within the cystic walls, ranging between 1–5 mm of new bony tissue. The mechanisms underlying bone regeneration following cycstic decompression have been intensively studied and it is thought that interleukin 1α (IL- 1α) might play an important role in this process. In odontogenic cysts, an intense inhibition of IL-1α expression was found after decompression associated with an important reduction in the size of the lesions [26]. Cystic decompression through marsupialization is defined as a conservative treatment method that has several advantages: it is an easy procedure that can be conducted under local anesthesia, requiring basic surgical equipment. Despite the simplicity, it is a highly tissue-sparing technique, allowing the preservation of bone height and anatomical structures, such as the inferior alveolar nerve or mental nerve [27,28]. Another important advantage of the procedure is its diagnostic character, allowing tissue sampling for histopathological assessment and diagnostic confirmation. In dental practice, cystic-appearing lesions represent a frequently encountered pathology, raising major diagnostic challenges for the clinician [29]. In order to provide the best therapeutic option, it is important to have a histological confirmation of the diagnosis [30]. Less invasive diagnostic procedures can be valuable in small lesions that can be radically treated by first intent [31]. Fine needle aspiration (FNA) has proven valuable, especially when associated with cell block preparation and hematoxolin–eosine staining, reaching 74.5% diagnostic accuracy compared to standard pathology in different jaw lesions. However, with regular cytology, which is usually performed after FNA, these rates drop to 56.8% accuracy compared to biopsy reports [32]. Another impediment for FNA is related to its limited availability in many countries. Thus, standard biopsy with pathology assessment of the specimen is the most used diagnostic tool today. An extensive study that collected data over an interval of 30 years reported that out of 44,000 specimens referred for pathology assessment from the maxillo-facial department, 13.8% were odontogenic cysts, thus confirming their high incidence in daily clinical practice. Other jaw-affecting pathology: non-odontogenic cysts, benign and malignant bone tumors, represented altogether 3.8% of the cases [33]. Therefore, even though the majority of the cystic-like lesions found in the oral and maxillofacial area are odontogenic cysts, clinicians always have to bear in mind the locally aggressive and destructive entities during the examination process. Bone lesions are frequently defined by similar clinical and imagistic characteristics that can lead to major diagnostic errors. Saghravanian et al. reported an accuracy rate of only 69.3% in patients that underwent surgery for clinically diagnosed odontogenic cysts after the specimen underwent pathology assessment [34]. A meticulous patient examination and history, with careful consideration for the site of the lesion, its borders, internal architecture, its effects on adjacent anatomical structures, generally can narrow the wide range of differential diagnostic options. In most cases, these lesions must be surgically removed and examined microscopically to establish an accurate diagnosis.

The Marsupialization technique implies the surgical creation of fenestration in one of the cystic walls and impediment of wound closure through specific sutures [35]; it reduces the hydrostatic pressure within the lesion and the surrounding bone with the direct consequence of blocking cystic growth and the underlying bone resorption. Subsequently, through a regenerative mechanism, fibroblasts migrate into the affected area and produce important quantities of collagenous matrix. These cells respond through complex signaling pathways by either proliferation or differentiation into a variety of cells, like bone cells, fat cells, or cartilaginous cells. The bone cells, osteoblasts, alongside osteoclasts, are involved in the process of bone healing through resorption, new bone apposition, and remodeling, leading to bone regeneration [36]. Moreover, the marsupialization technique should be taken into consideration as a primary approach in patients with erupted or impacted teeth involved in the cystic area in order to preserve the teeth and therefore improve the functional and aesthetic outcomes [37,38]. For maxillary lesions, an alternative decompression method using plastic tubes within the cavity has revealed great results in cystic size reduction [39].

The main drawbacks of marsupialization are related to the incomplete removal of pathological tissue, which can be associated with higher rates of recurrence, as well as the prolonged time of healing [40]. Considering these limitations, decompression can be used as an intermediate step in the treatment of large cystic lesions, with the intention of reducing the cystic dimensions rather than a definitive treatment alone. It is then followed by radical excision, with or without additional reconstructive procedures, thus minimizing the risk of recurrence [41]. Therefore, any cystic lesion that does not jeopardize the integrity of the mandible after surgical removal should to be treated radically by first intent whenever possible. Radical surgical excision with complete removal of the cystic capsule is the most important element for a predictable result [42]. Another potential risk when using marsupialization as a stand-alone treatment is the malignant transformation of the residual cystic membrane in time [43,44]. In our case, radical cystic excision was conducted half a year after marsupialization, with the addition of a titanium mesh reconstruction covering the mandibular fenestration, in order to prevent dehiscence with subsequent chronic oro-cystic fistula. At the one-year follow-up visit, the patient showed no history or signs of impaired healing in the cystic area and the procedures for dental arch restoration were initiated. The use of titanium mesh in the final surgery allowed optimal bone formation and soft tissue healing, without the necessity of bone grafting with autologous bone grafts, which would imply more complex surgeries with potential donor site morbidity, and no additional costs associated with different market-available grafting materials.

## 4. Conclusions

Large odontogenic mandibular cysts imply a meticulously conducted clinicopathological assessment and treatment. Marsupialization should be taken into consideration in the treatment planning of large cystic lesions as an initial, less invasive, surgical step that leads to secondary radical enucleation, with minimal risks for the patient. Guided by the same principles of safety and simplicity, the healing process can be optimized through the use of titanium meshes, as an alternative for other reconstructive techniques, in the management of large cystic lesions.

## Data Availability

The data used and/or analyzed during the present study are available from the corresponding author.
